# Three-dimensional fine crust-mantle structure imaging and structural characteristics of Bohai Sea

**DOI:** 10.1038/s41598-023-51101-4

**Published:** 2024-01-09

**Authors:** Yong Ma, Lei Gao, Demiao Dong

**Affiliations:** 1 Tianjin Earthquake Agency, Tianjin, 300201 China; 2 Chinese Academy of Geological Sciences, Beijing, 100037 China

**Keywords:** Geophysics, Seismology

## Abstract

This study used the consistency-constrained double-difference tomography method to invert 3D fine structure models of Vp, Vs, Vp/Vs at depths above 60 km, and precise relocation parameters of earthquakes in the Bohai Sea. According to the results, the velocity structures of P-wave and S-wave in the Bohai Sea area were highly similar and demonstrated noticeable lateral non-uniformity. The crust beneath the Tanlu Fault displayed a clear stratification structure, with a continuous velocity transition in the middle of the crust. The intricate crustal structure beneath the Zhangpeng Fault displayed high-velocity bodies in the crust and low-velocity anomalous zones connected to the top of the mantle in the lower section of the crust. The structural pattern in the deep crust of the Bohai Sea controls the occurrence characteristics of the Zhangpeng Fault and the Tanlu Fault. The earthquakes in the Bohai Sea area were concentrated mainly in the southern part near the Zhangpeng Fault. There is a good correspondence between the relocated earthquakes and velocity structure. There are many significant differences in crustal structure between the north and south of the Bohai Strait, and there are obvious velocity anomalies in the middle and upper crust. The distribution shape of high Vp/Vs value indicates that mantle material migration has occurred at the bottom of the crust. This paper provided important reference for further research on the relationship between deep tectonic features and tectonic activity in the North China Craton.

## Introduction

The Bohai Sea is an inland depression in North China, surrounded by "Three Bays and One Strait" (Liaodong Bay, Bohai Bay, Laizhou Bay and Bohai Strait). Geomorphologically, the Bohai Sea is the extension of the North Huanghai Sea into the interior of North China. Geotectonically, the Bohai Sea an important part of the Bohai Bay basin in China. Meanwhile, the Bohai Sea, as the intersection of the north-east-oriented Tanlu Fault and the north-west-oriented Zhangpeng Fault, has complex regional tectonics and strong seismic activity as one of the most seismically active regions in the eastern part of China^1,2^. Since 1500, there have been a total of 72 earthquakes with a magnitude of 5 or above in the Bohai Sea area, including 7 earthquakes with a magnitude of 7 or above and 17 earthquakes with a magnitude of 6 or above (China Earthquake Networks Center). This region has been the hot spots for research on geodynamics, strong earthquake breeding environment with earthquake prediction and forecasting due to the unique tectonic background and frequent and violent seismic activity. For decades, in order to understand the internal structure of the crust in this area, geophysical fields such as gravity field^3^, geomagnetic field^4,5^, geothermal field^6^ and artificial seismic bathymetry^7–9^, body wave travel time tomography^10–13^, surface wave laminar imaging^14,15^, and receiver function^16^, and other seismology imaging inversion methods have conducted a lot of research on the characteristics of crustal structure and geophysical characteristics in a large range in this region.

The 3D seismic tomographic imaging method is the most intuitive and effective research tool to obtain the structure of the subsurface medium. However, due to the limitations of seismic data and research methods, most of the existing research results focus on the land area around the Bohai Sea, and only a few involve the Bohai Sea area, whose results can only provide large scale velocity structure images, which cannot distinguish the local small-scale fine structure. To this end, this study uses a large amount of seismic arrival data accumulated by the seismic monitoring network in and around the Bohai Sea to jointly invert the high-resolution Vp, Vs and Vp/Vs three-dimensional crustal structure models by using the consistency-constrained double-difference tomography method^17^. This method is a further improvement on the original double difference tomography method. It not only obtains accurate source position parameter information and Vp, Vs models by constructing seismic pairs, but also obtain more accurate 3D Vp/Vs models by selecting appropriate damping factors and smoothing weights. The research result can provide important scientific references for understanding the deep structure, focal mechanism, and tectonic evolution of the Bohai Sea.

## Data and method

**Figure 1:**
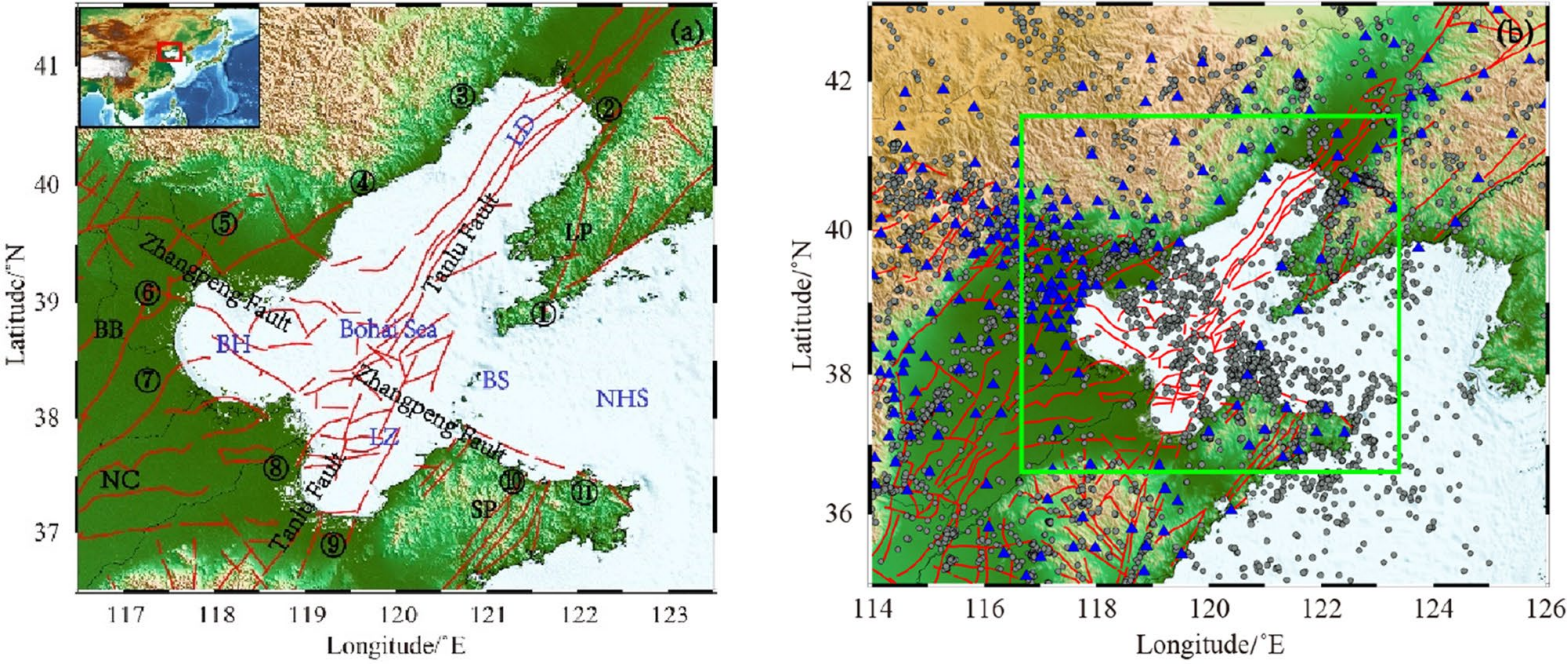
Distribution map of faults, earthquakes and stations in the study area. These figures were created using GMT v5.4.4 software. (**a**) Shows the structural map of the study area. The red curve represents the main fractures within the region. The letter is the abbreviation of the region name, in which NC is North China Plain, LP is Liaodong Peninsula, SP is Shandong Peninsula, LD is Liaodong Bay, BH is Bohai Bay, BB is the Bohai Bay Basin, LZ is Laizhou Bay, BS is Bohai Strait and NHS is the North Huanghai Sea. The numbers represent the city name, where ① is Dalian, ② is Yingkou, ③ is Huludao, ④ is Qinhuangdao, ⑤ is Tangshan, ⑥ is Tianjin, ⑦ is Cangzhou, ⑧ is Dongying, ⑨ is Weifang, ⑩ is Yantai, and ⑪ is Weihai. (**b**) Shows the distribution of research data. The gray dots represent the initial positions of the earthquakes involved in the inversion. The blue triangle represents the seismic observation station. The green box represents the imaging range of this study.

**Figure 2:**
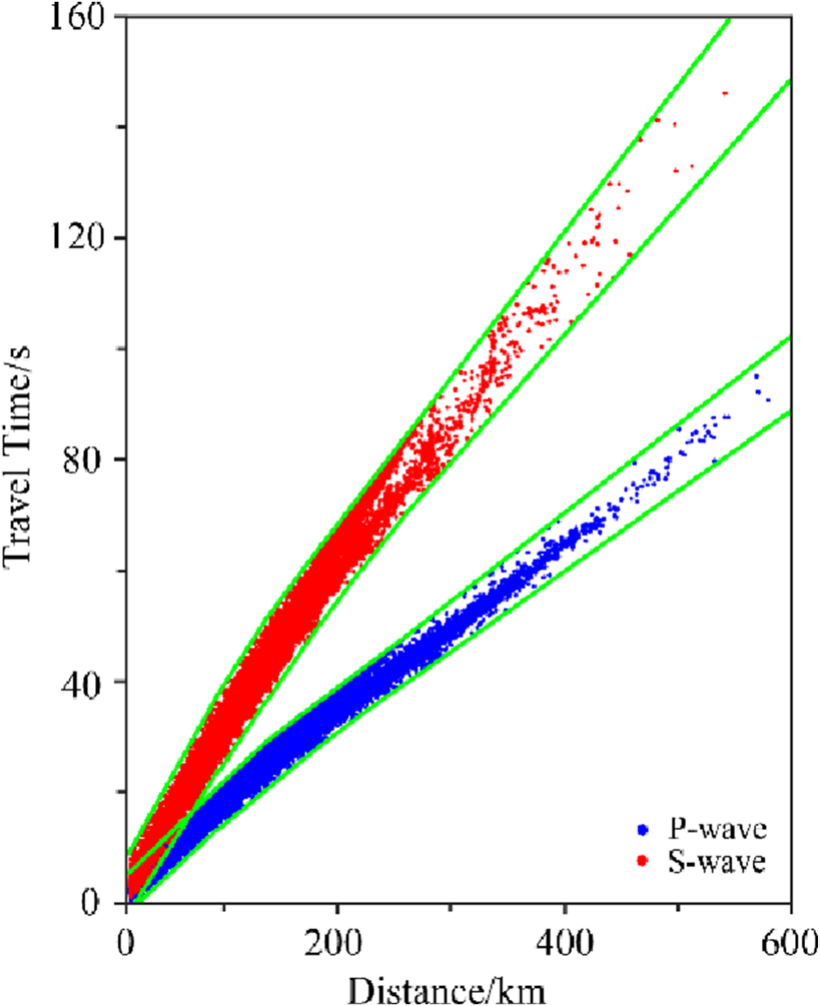
Curves of P- and S-wave travel times.

This study selected the range of 116.5–123.5°E and 36.5–41.5°N as the imaging study area (Fig. 1a), and collected the P- and S-wave arrival data of M_L_ ≥ 1.0 natural seismic events recorded by 200 stations in the range of 114.0–126.0°E and 35.0–43.0°N from January 2008 to January 2022 at the China Seismological Network Center (Fig. 1b). To mitigate the influence of anomalous data in the calculation process and improve the accuracy of the inversion results, all seismic phase data with large deviations in the travel-time curves were deleted (Fig. 2). According to the distribution of earthquakes in the study area, the maximum distance between pairs was set to 10 km, each earthquake was recorded by at least 5 stations in the study area, each pair could be formed with at most 30 earthquakes. Finally, 139,601 P-wave and 141,309 S-wave absolute arrival data of 7734 earthquakes were selected to meet the requirements, which fully covered the ray paths in and around the Bohai Sea.

The double difference seismic tomography method^18^ skillfully constructs seismic pairs from different earthquakes recorded by the same observation station k and inverts the velocity structure of underground media by using the difference between the observed travel time and theoretical travel time residuals of different sources *i* and *j*:

1 $$dr_{k}^{ij} = (T_{k}^{i} - T_{k}^{j} )^{obs} - (T_{k}^{i} - T_{k}^{j} )^{cal} = \sum\limits_{m = 1}^{3} {\frac{{\partial T_{k}^{i} }}{{\partial x_{m}^{i} }}} dx_{m}^{i} + d\tau^{i} - \sum\limits_{m = 1}^{3} {\frac{{\partial T_{k}^{j} }}{{\partial x_{m}^{j} }}} dx_{m}^{j} - d\tau^{j} + \int\limits_{i}^{k} {\delta uds} - \int\limits_{j}^{k} {\delta uds} ,$$

where $dr_{k}^{ij}$ represents the double difference, $T_{k}$ is the initial seismogenic time of the source, $dx_{m}$ is the disturbance of the source position parameter, $d\tau$ is the disturbance of the earthquake occurrence time, $\delta u$ is the slowness of seismic wave, and $ds$ is the path integral. Compared with the traditional body-wave travel time imaging, this method not only uses the absolute arrival time of seismic wave in the propagation process but also adds the relative arrival time information of seismic pair, which can effectively reduce the influence caused by the abnormal velocity structure on the ray path from source to station and obtain accurate and reliable 3-D Vp, Vs structural models, and seismic relocation results^19^. On this basis, Zhang et al.^20^ introduced the linear relationship^21^ between the time difference (S–P) of the P- and S-waves of the same earthquake similar path and the seismic position disturbance and Vp/Vs on the ray path into double difference tomography. Through the joint inversion of the relative and absolute arrival time of P-, S-wave, and S–P between seismic pairs, the 3-D velocity models and relocation results of Vp, Vs, and Vp/Vs were obtained. However, two different Vp/Vs models can actually be obtained in accordance with the method^17^. The first step was to divide the Vp model by the Vs model, but given the large S wave data error and less S-wave data, the Vs model was more difficult to distinguish than the Vp model, resulting in greater uncertainty of the Vp/Vs model. The second step involved the use of the linear relationship between the P-, S-wave arrival time difference (S–P) of the same path and seismic position disturbance, and Vp/Vs on the ray path^21^. The travel time difference of S–P was introduced into double-difference tomography and obtained by direct inversion. However, a part of S–P data was eliminated to meet the requirement of ray path similarity between P- and S-waves. Thus, the Vp/Vs model was extremely reliable, but the resolution was relatively reduced. Usually, given the different calculation methods of Vp/Vs model, the results of the two models differed^17^.

In response to this phenomenon, Guo et al.^17^ established a consistent constraint relationship between the two Vp/Vs models:

2 $$\Delta = k_{1} - k_{2} = \frac{{u_{s} }}{{u_{p} }} - k_{2}$$

where $k_{1} = \frac{{u_{s} }}{{u_{p} }}{ = }\frac{Vp}{{Vs}}$ represents the Vp/Vs model obtained by dividing Vp by Vs, $u_{p}$ and $u_{s}$ denote the slowness of the P- and S-waves, respectively, $k_{2}$ corresponds to the Vp/Vs model obtained by the inversion of S–P data, and ∆ is the difference of the two Vp/Vs models. The relationship between the true and calculated values was converted to a linear correlation of $u_{p}$, $u_{s}$, and $k_{2}$ using a truncated Taylor expansion,

3 $$d\Delta = \Delta^{obs} - \Delta^{cal} = k_{2} - \frac{{u_{s} }}{{u_{p} }}{ = }\frac{\partial \Delta }{{\partial u_{p} }}\delta u_{p} + \frac{\partial \Delta }{{\partial u_{s} }}\delta u_{s} + \frac{\partial \Delta }{{\partial k_{2} }}\delta k_{2} = - \frac{{u_{s} }}{{u_{p}^{2} }}\delta u_{p} + \frac{1}{{u_{p} }}\delta u_{s} - \delta k_{2}$$

Similar to the traditional double-difference tomography method, the consistency-constrained double-difference tomography method can be determined a Vp/Vs model with good resolution and high reliability by selecting appropriate damping factors and smooth weights. Thus, this method can obtain not only accurate 3-D P- and S-wave velocity structures but also accurate 3-D Vp/Vs models^17^.

## Calculations and results

**Figure 3:**
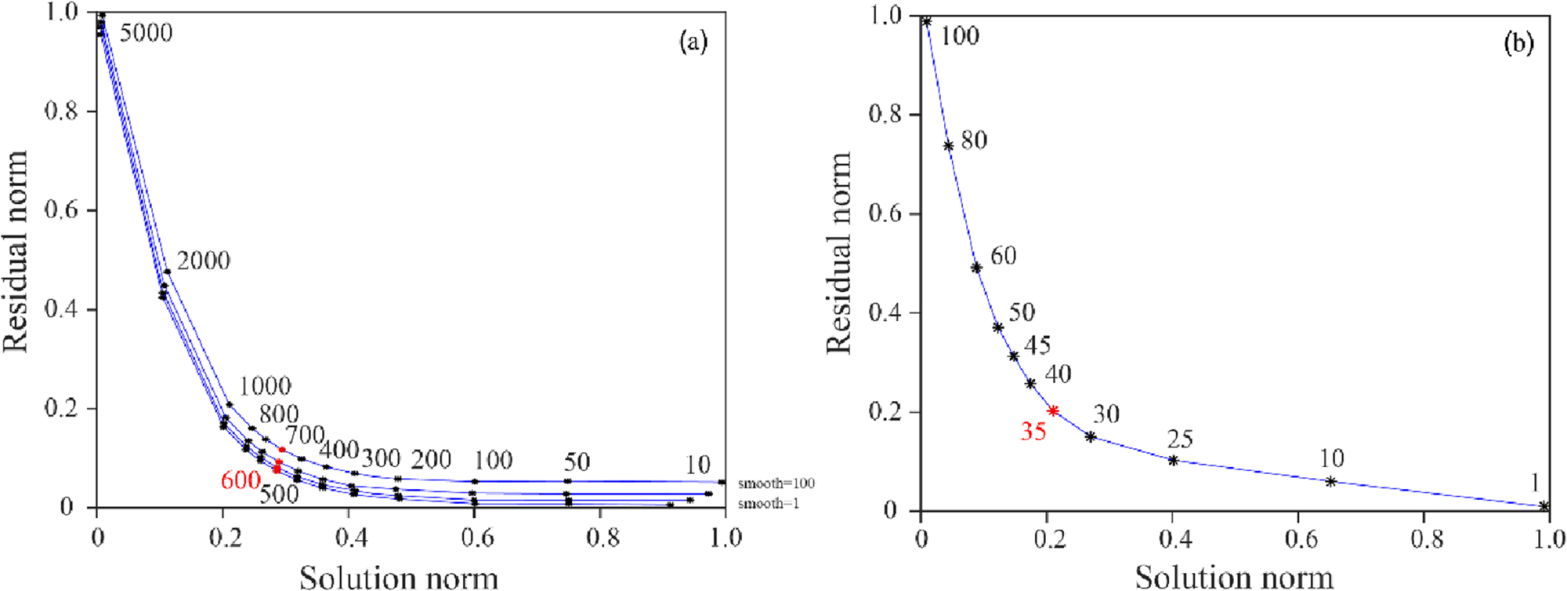
Trade-off curves of damping parameters and smoothing weights. (**a**) Shows the trade-off analysis between the normalized solution norm and data residual norm from inversions with a set of smoothing and damping parameters. Different curves represent disparate smoothing values of 1, 10, 50, 100 and 150. The optimal damping parameter is determined to be 600. (**b**) Shows the trade–off analysis between the normalized slowness norm and the data residual norm for a set of smoothing parameters using the optimal damping parameter of 600. The optimal smoothing parameter is selected 35.

The initial velocity model for the inversion is the research result of joint inversion of body-wave and surface-wave in Chinese Mainland by Han et al.^22^. The velocity values at different depths are obtained through linear interpolation^23^. According to the distribution of earthquakes and stations in this area, the resolution test of different grid spacing was carried out. The horizontal grid spacing was 0.5° × 0.5°, and the depth grid nodes were 0, 7, 14, 21, 28, 35, 45 and 60 km. In the damped LSQR algorithm, damping configuration and smooth weight parameters directly affected the final inversion result. The single-iteration variance equilibrium curves with different damping factors and smoothing weights were drawn to obtain more stable results. Referring to the selection rule of the optimal solution of equilibrium curve and given the relationship between normalized solution norm and data residual norm, the damping of the first inversion was set to 600, and the smoothing weight parameter was 35 (Fig. 3).

**Figure 4:**
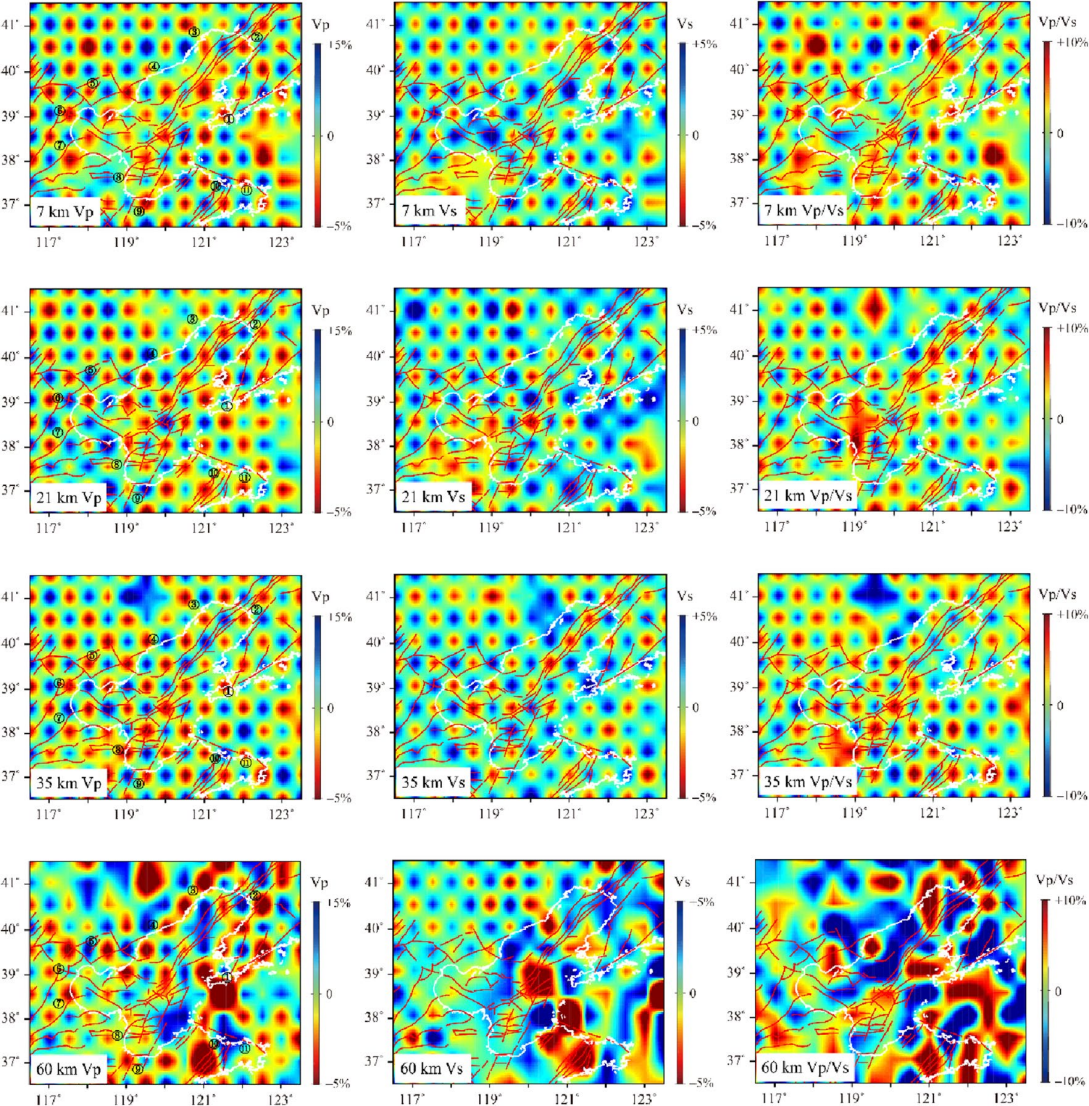
Different depth checkerboard test results in Bohai Sea. These figures were created using Matlab R2017a software. The white curve represents the coastline, and the numbers in Vp represent the city name.

Using the detection board method proposed by Zhao et al.^24^, forward calculation of the travel time data set in the initial velocity model was carried out based on the actual source and station location information. Then, the synthetic data set with adding 5% random noise was inverted using the consistency-constrained double-difference tomography method, and the inversion result was utilized as the reconstruction model. The resolution capability of the inversion data model was tested by comparing the values of each node in the initial and reconstructed models. Figure 4 showed the results of the check board test at different depths in the Bohai Sea region. In the North Huanghai Sea area, it is less resolvable due to the sparse distribution of stations. Due to the influence of ray paths, the resolution ability of the Dongying area in the range of 7–21 km and the northern part of Qinhuangdao in the depth of 28–35 km was slightly weaker than other areas. At 60 km depth, the resolution of Vp, Vs, and Vp/Vs became gradually weaker in the central Bohai Sea region due to the small number of earthquakes in the deep part of the crust, the inversion results could be regarded as a good reference since the initial model is the result of previous 3D research in this region. Except for this, every node in other areas of the study region at different depths could be recovered perfectly. The detection results could meet the requirements of studying the 3D crust-mantle structure in the Bohai Sea through the checkerboard grid resolution test, with real and reliable inversion results, which had important reference value.

**Figure 5:**
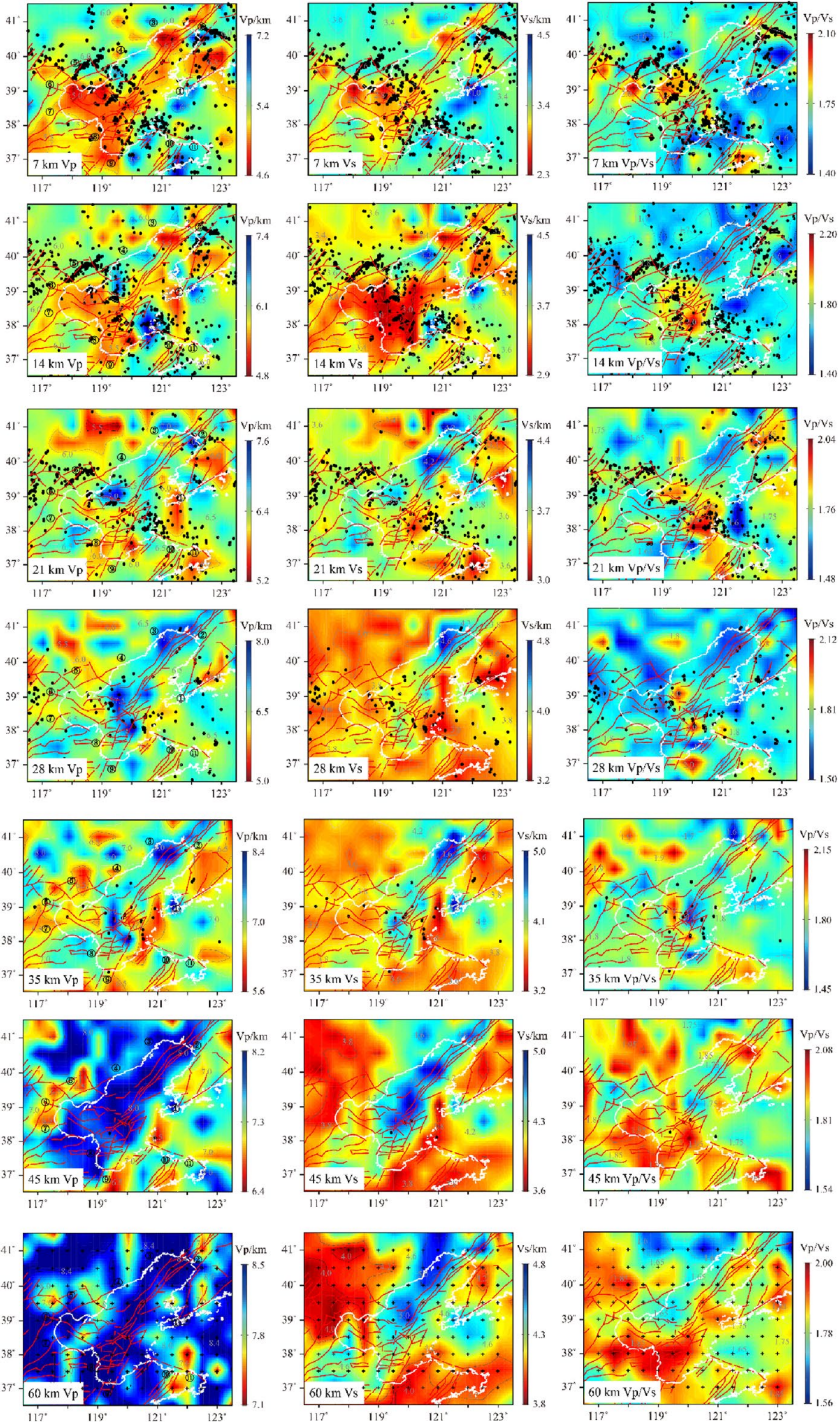
Vp, Vs, Vp/Vs and the relocated earthquake distribution within the interval between layers on both sides of the profile in Bohai region. These figures were created using Matlab R2017a software. The black dots indicate the projection of the relocated earthquakes. The red curves indicate the main faults. The white curves indicate the coastline, the black numbers in Vp represent the city name, and the black crosses represent the parameterization nodes of the model.

After 20 iterations, the travel time residual significantly decreased. The root mean square of travel time residual did not change. Finally, the accurate relocation information of the participating inversion earthquakes and stable Vp, Vs, and Vp/Vs results in the Bohai Sea area was obtained (Fig. 5). Compared with the initial seismic, the epicenter location of the relocated seismic in the Bohai Sea area had more concentrated seismic distribution. The imaging results showed that the Vp and Vs structures had good similarity in most areas, but the inversion results also showed some significant differences in some areas due to the different propagation characteristics of P and S waves in the medium. To discuss the relationship between seismic distribution and velocity structure in the study area, we projected the precise locations of relocated earthquakes onto the nearest horizontal velocity structure profiles, respectively. At a depth of 7 km in Fig. 5, there was a good correspondence between the velocity structure distribution and the topographic features and fault orientation. In the Bohai Sea, Vp and Vs were obvious low velocity anomalies below the Tanlu Fault and the Zhangpeng Fault and the area to the south. P-wave showed low velocity characteristics in the Liaodong Peninsula and high velocity characteristics in the Shandong Peninsula. Vp/Vs were obvious high value anomalies in the sea near Tangshan and the southern part of the Shandong Peninsula. At 14 km depth, the low velocity range of P-wave decreased, the high velocity anomaly was obvious in the northern part of the Zhangpeng Fault, and there were low velocity anomalies in the Bohai Strait. The range of low velocity anomalies of S-wave expanded, and the high velocity anomalies were only in the two ends of the Bohai Strait and the eastern part of Qinhuangdao. The high value of Vp/Vs was shifted to the northeast of Yantai. At 21 km depth, both Vp and Vs maps showed an increase in the high velocity body and a decrease in the range of low velocity body. In the area where the Tanlu Fault and the Zhangpeng Fault intersected, there were obvious differences in the velocity structure distribution between the north and south sides. The area of high value of Vp/Vs was expanding in the former northeast Yantai. At 28–45 km depth, the Vp and Vs high velocity areas below the Tanlu Fault gradually increased until most of the Bohai Sea. The Vp/Vs high value area increased with depth in the southern Bohai Sea and the northern area north of Qinhuangdao and Huludao. Without varying Vp/Vs values, the high velocity was dominant in all other areas except for the low velocity anomalies at some locations. There was only one obvious high value anomaly in the eastern part of Bohai Bay in Dalian.

## Discussion

**Figure 6:**
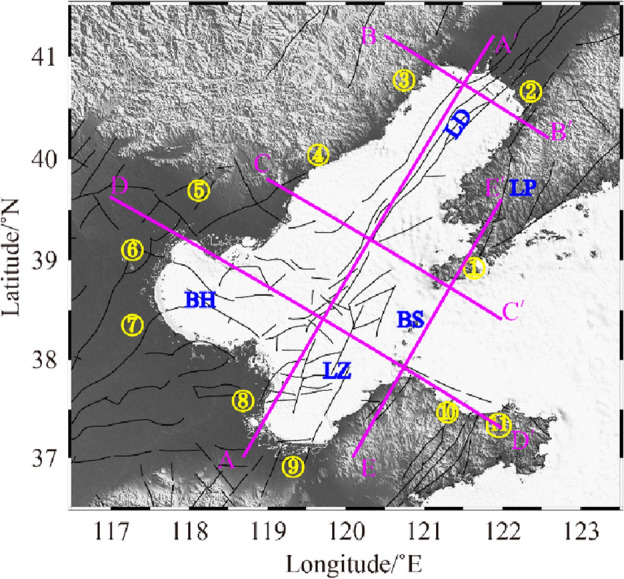
The distribution map of vertical profile positions. This figure was created using GMT v5.4.4 software.

The Bohai Sea is a land margin bay in eastern China, belonging to the Cenozoic rift system on the eastern edge of the North China Plain, and the overall structure is NNE-oriented. The Cenozoic sedimentary layer is generally above 6000–12,000 m^25^. The Bohai Sea region is highly developed faults, with intersecting north-east-trending Tanlu Fault and the north-west-trending Zhangpeng Fault, while branching faults in different directions intersecting each other to form a complex tectonic system^26,27^. The southern part of the Bohai Sea and the Laizhou Bay area are strongly influenced by the Zhang-Peng Fault, accompanying small frequent earthquakes, with moderate and strong earthquakes occurring from time to time^28^. In contrast, the northern Liaodong Bay area has less seismic activity and almost no moderate or strong earthquakes, showing distinctly different seismic activity characteristics in the north and south regions^11^. The distribution of earthquakes in the Bohai Sea region in the past decade is consistent with the historical seismicity pattern in the region, also the relocated source locations of the earthquakes in the southern Bohai Sea have multiple seismic dense areas, and the source depths are mostly concentrated in the range of 5–25 km. Velocity structure images of horizontal profiles at different depths (Fig. 5) show that the velocity structure of P and S waves in the Bohai Sea has good consistency. In the upper crust, it shows a low velocity anomaly^13^, and the velocity of the crustal medium gradually increases with depth, which is in contrast with the surrounding area. At 7 km depth, except for the Liaodong Peninsula, the velocity distribution corresponds to the topographic features at the shallow surface, with the mountainous areas of the land corresponding to the high-velocity anomaly and the depressional zone and sedimentary basin corresponding to the low-velocity anomaly. At 7 km and 14 km depth (Fig. 5), the low velocity anomalies of Vp and Vs in the Bohai Sea are concentrated below the Zhangpeng Fault and the Tanlu Fault, and the distribution of velocity structures in the crust controls the occurrence characteristics of these two faults. Starting from 21 km depth, significant high velocity anomalies in Vp and Vs appear simultaneously in the northern part of the intersection of the two faults. Until 35 km depth, this high-velocity body gradually expands southward to the sea of Laizhou Bay, which indicates that the heterogeneity of seismic activity in the Bohai area is related to the crustal structure, also the complex crustal structure in the southern Bohai Sea and the Laizhou Bay area is one of the important reasons for the frequent occurrence of seismic activity. In the range of 45–60 km, the high velocity body expands almost to the whole Bohai Sea, with smaller difference between the P-wave velocity and the surrounding areas of the Bohai Sea, but the Vs at 60 km depth has a large difference with the surrounding areas, thus highlighting the high velocity anomaly in the Bohai Sea area. There are two obvious high-value points of Vp/Vs between 7 and 35 km depth in the Bohai Sea, which are located in the north of the intersection of the Zhangpeng Fault and the Tanlu Fault and in the east of the seismic active region in the Yantai area. At depths of 45 km and below, the magnitude and distribution differences of Vp/Vs keep decreasing, which have synchronous variation characteristics with the velocity structure in this depth range. In order to have a clear understanding of the crust-mantle structural characteristics in the Bohai region, we conducted detailed analysis of vertical profiles in different directions in some key areas (Fig. 6).

**Figure 7:**
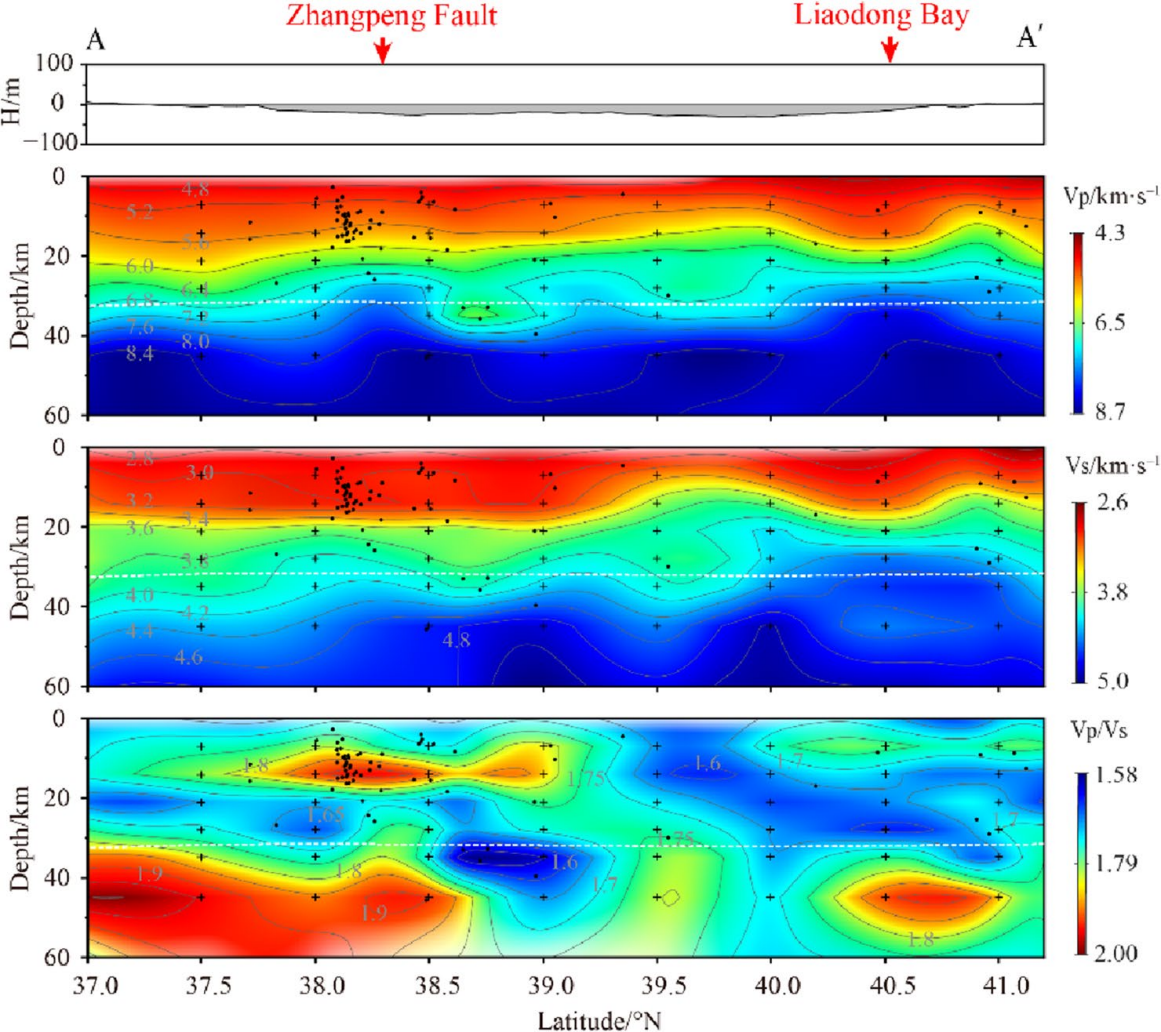
Vp, Vs and Vp/Vs vertical section structural images of profile A (the section position is shown in Fig. 6). Black dots represent projections of relocated earthquakes within 10 km on both sides of the profile. The black crosses represent the parameterization nodes of the model. White dotted lines represent the Moho surface in the Crust 1.0 model.

The Tanlu Fault is a large-scale tectonic zone in eastern China, has undergone tectonic deformation and magmatic activities such as sliding, stretching, squeezing since the Mesozoic^29,30^. The Bohai Sea is a key area sections of the Tanlu Fault, and the structural characteristics of the crustal velocity beneath the fault have been the focus of geological research. Figure 7 shows a vertical section along the strike of the Tanlu Fault (profile A). The Vp and Vs imaging results in the figure show that the crust near the Tanlu Fault has an obvious stratified structure. In the area of above 15 km, there are low velocity bodies with large thicknesses, thus indicating a giant thick Cenozoic sedimentary cover in the Bohai Sea, which is consistent with the results of previous studies^15,31^ on the shallow structure in this area. There is a continuous velocity transition within the range of 15–20 km. The seismic wave velocity gradually increases under the transition zone, and little velocity variation along the trend of the Fault^11^. At depths below 30 km, Vp and Vs results show different distribution characteristics. The images (Fig. 7) show that Vp starts to appear intermittent high-velocity anomalies synchronously at 40 km, while Vs only shows high-velocity anomalies in the central Bohai Sea and Liaodong Bay area. The Vp/Vs along the direction of the Tanlu Fault show obvious high values at the top of the upper mantle, and there are horizontally distributed high value layers in the intersection area with the Zhangpeng Fault in the interior of the crust, while all other areas are dominated by low values.

**Figure 8:**
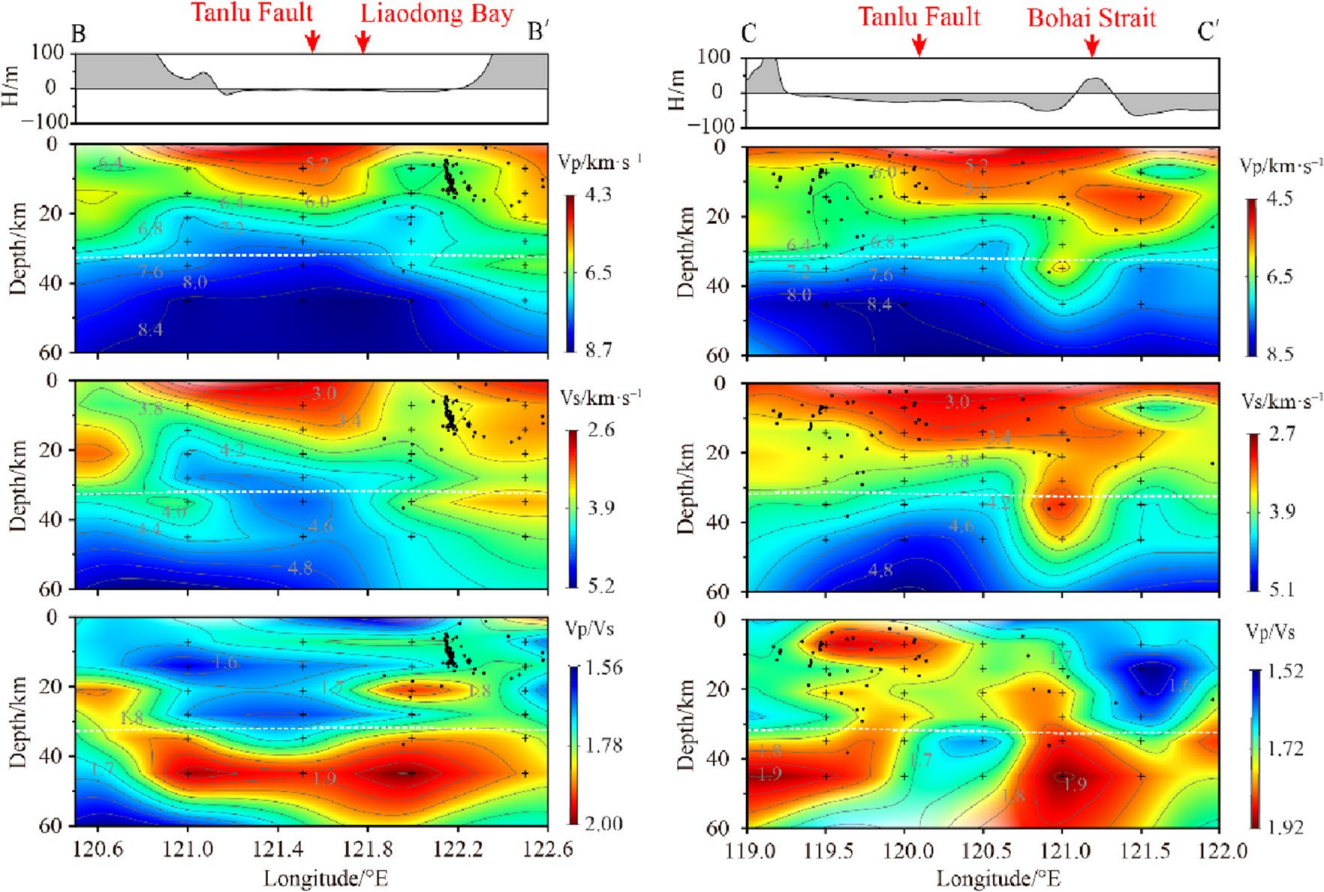
Vp, Vs and Vp/Vs vertical section structural images of profile B and C (the section position is shown in Fig. 6).

B, C and D are three depth profiles perpendicular to the direction of the Tanlu Fault. Profile B (Fig. 8) is located in the Liaodong Bay area in the northern section of the Tanlu Fault, and the low-velocity anomalies within the crust in the figure correspond to the Liaodong Bay area, while the high-velocity bodies on both sides correspond to the hilly areas on land. The seismic activity area is located on the transition zone of the high and low velocity bodies on the side of the high velocity body, which slopes downward to the deep part of the crust at a high angle along the direction of the velocity transition zone. Profile C (Fig. 8) starts from the left in Qinhuangdao area, crosses the central Bohai Sea and the Bohai Strait, and enters the Huanghai Sea. The crustal velocity at the location of the Tanlu Fault shows a sharp change, and the maximum depth extends to 20 km below the surface, which supports the fault occurrence characteristics of previous works^32–34^ that it is steeply dipping and may extend to the deeper part of the crust. The upper part of the crust still shows a thicker low-velocity layer and the thickness gradually increases from left to right, which is similar to the change in seafloor elevation. However, in Bohai Strait and the area to the east, the upper crust shows a high velocity body, and the middle and lower crusts are a huge wedge-shaped low velocity body extending eastward under the Bohai Sea with a low velocity strip deep to the top of the mantle. The high-velocity body on the right side of the section corresponds to the Northeast high-velocity zone in the horizontal section (Fig. 5). There is a NW trending tectonic stress during the movement of the Pacific plate and the Philippine Plate towards the Eurasian plate^35^. The high-velocity areas perpendicular to the stress direction must bear stronger regional tectonic stress.

**Figure 9:**
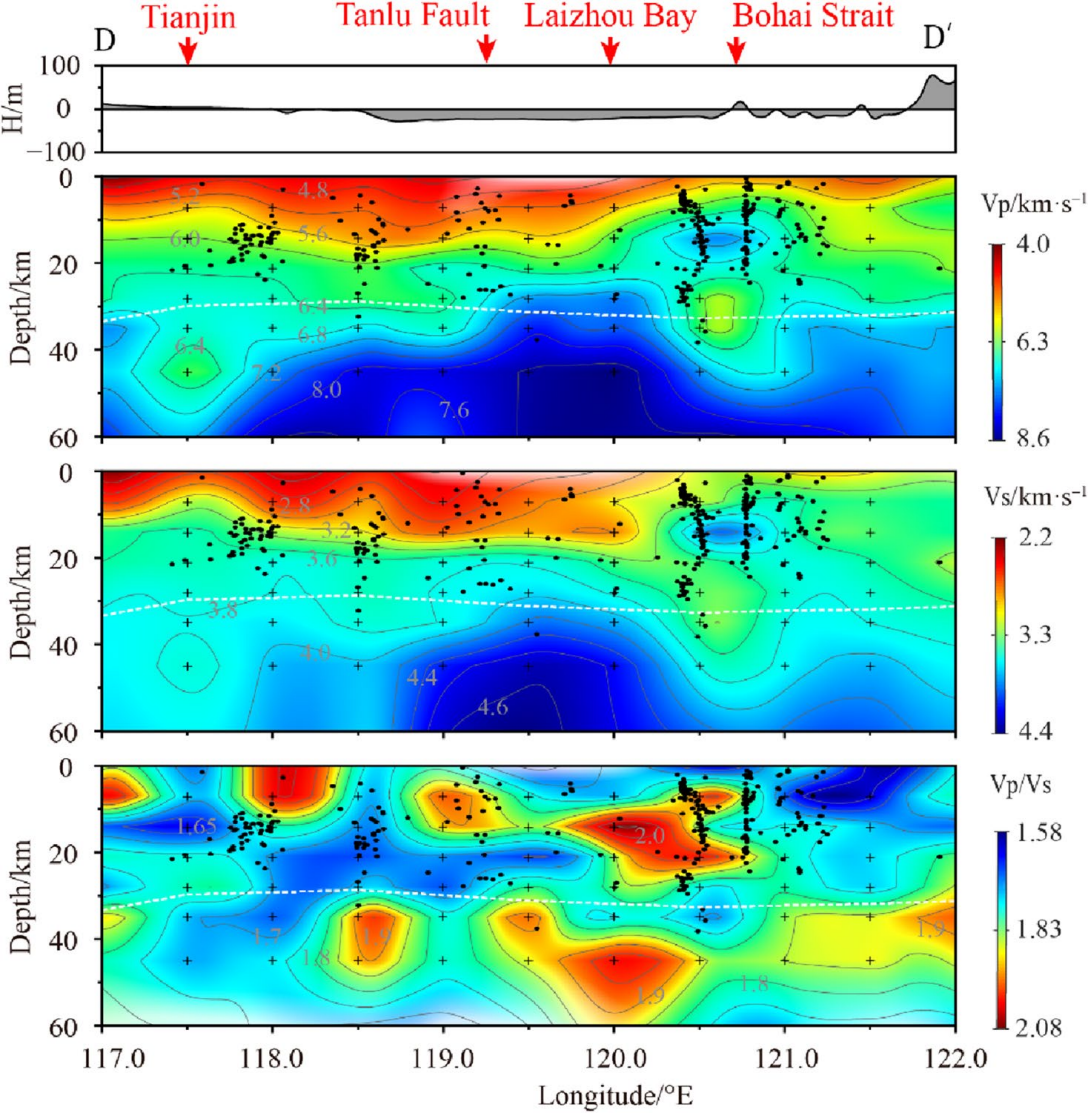
Vp, Vs and Vp/Vs vertical section structural images of profile D (the section position is shown in Fig. 6).

The Zhangpeng Fault is a very important tectonic zone in the southern Bohai Sea, with a width of 18 km, a series of basement faults, a complex crustal structure, and frequent seismic activity^12^. Profile D (Fig. 9) runs through the southern part of the Bohai Sea and the Bohai Strait along the Zhangpeng Fault. The relocated earthquakes have obvious clustering phenomenon. Most of the source locations are concentrated on the side of the high velocity body or the high and low velocity transition zone near the high velocity body. Due to the different structures of the seismic source areas, there are slight differences in earthquake distribution. In the central Bohai Sea, earthquakes are mainly concentrated in the middle of the crust at a depth of 10–20 km. Near the Bohai Strait, earthquakes have significant activity in the range of 0–30 km. From the seismic projections within 10 km on both sides of the profile, it can be seen that earthquakes in the Yantai area have obvious vertical distribution characteristics, with the deepest position extending along the edge of the lower crustal low-velocity body to the top of the upper mantle, and the continuous intensive seismic activity outlines the occurrence characteristics of the fault. Based on the imaging results of the vertical section (Fig. 9), it is easy to see the complex velocity structure beneath the Zhangpeng Fault, with not only large lateral variations, but also obvious high-velocity anomalies within the crust^7^. In the upper part of the crust, the distribution patterns of Vp and Vs are basically similar. The left onshore area and the southern Bohai Sea area are thicker low-velocity layers, while the low-velocity layers of the sea area east of the Bohai Strait are thinned or even disappeared and replaced by obvious high-velocity bodies. Between the lower crust and the top of the mantle (depth 28–35 km), the high velocity in Laizhou Bay area is relatively high, and gradually decreases to both sides. The P-wave velocity structure image (Fig. 9) shows that there are two low velocity anomalous channels below the Tianjin and the Bohai Strait. According to the findings of existing results^36,37^, it is interpreted that the lower velocity areas in the lower crust and upper mantle are related to the upwelling of mantle material.

**Figure 10:**
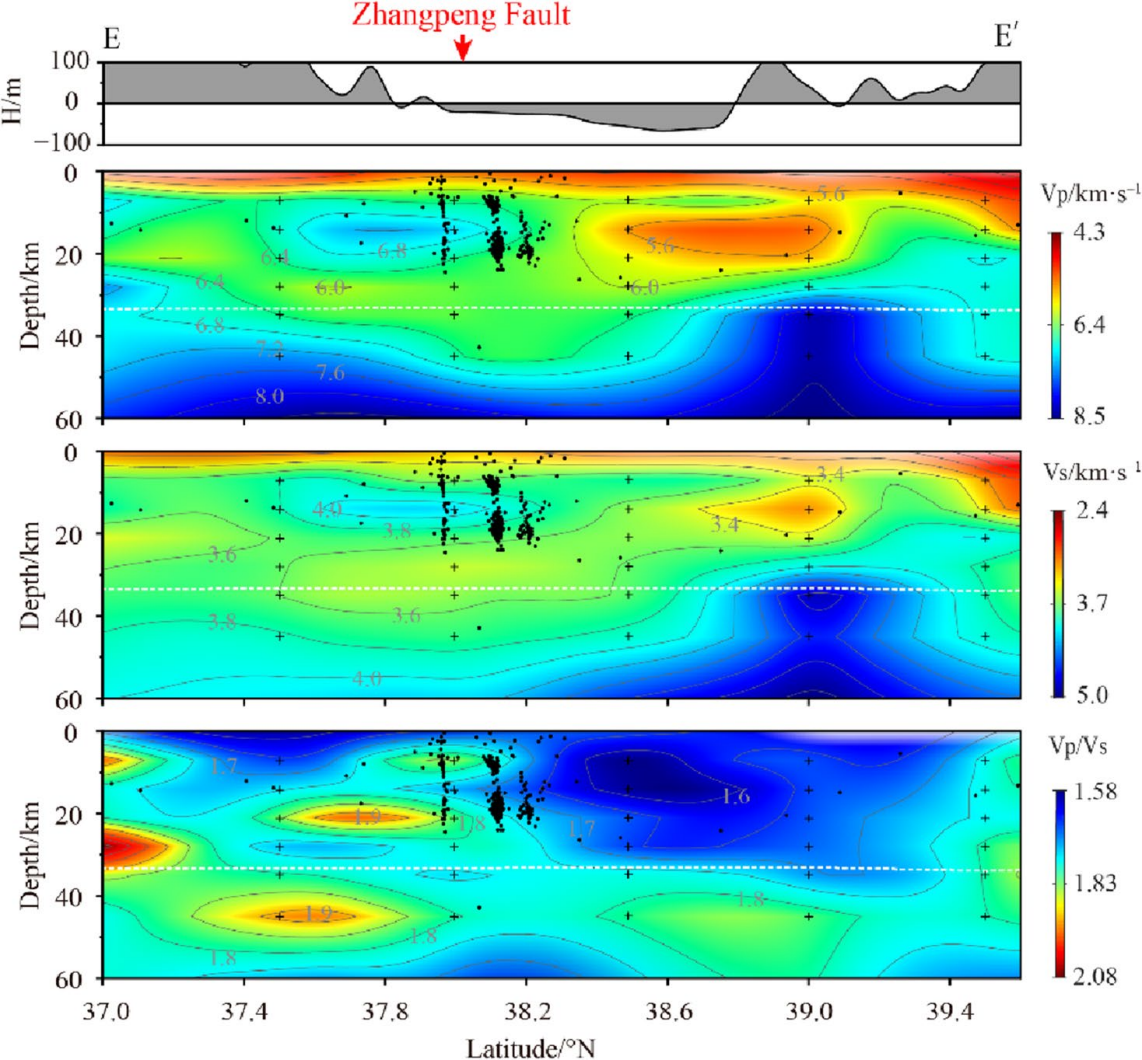
Vp, Vs and Vp/Vs vertical section structural images of profile E (The section position is shown in Fig. 6).

The Bohai Strait is located on the eastern part of the Bohai Sea, which is the dividing line between the Bohai Sea and the North Huanghai Sea tectonically controlled by the Tanlu Fault and the Zhangpeng Fault. It was uplifted under the collision and extrusion of the Pacific plate and the Eurasian plate^38^. Profile E (Fig. 10) shows a vertical section along the Bohai Strait. Compared to the Bohai Sea, the low-velocity layer at the top of the strait crust is thinned, and the velocity structure within the crust exhibits significant non-uniformity. The low-velocity anomalous bodies present in the crust are relatively soft and have strong plasticity. When the tectonic stress in the study area changes, the low-velocity layer in the crust will transform the regional stress into strain energy or transmit it to the surrounding high-velocity body through deformation^39,40^. The scarcity of seismic activity in the northern part of the Bohai Strait may be attributed to the presence of low-velocity anomalous bodies within the crust. There is a high-velocity body in the middle crust in the southern part of the strait. Seismic activity is frequent near the high-velocity bodies, with the depth of the source between 5 and 20 km depth in a vertical band (profile E). Most earthquakes are concentrated at both ends of each strip. Few earthquakes are near the depth of 12 km corresponding to the high-velocity abnormal body in the middle of the strip. It is provided supporting evidence that this particular high-velocity body is weak property. In the southern part of the Bohai Strait, the low-velocity characteristics in the lower crust below the Zhangpeng fault zone are very obvious. It separates the high-velocity block inside the crust and the upper mantle, which increases the thickness of the crust-mantle transition zone. Previous authors^11,12,41^ suggest that the low-velocity layer was caused by multiple periods of magmatic activity in the Mesozoic and Cenozoic eras, and was related to the bottom intrusion during the destruction of the North China Craton and the extension of the lithosphere. Since the depth of 60 km (profile E), the high value area of Vp/Vs extending to the upper left again indicates that the low-velocity interlayer at the bottom of the crust in this area may have been caused by the uplift of the asthenosphere or the local change of heat flow activity.

Petrophysical experiments have shown that Vp/Vs is an important means of studying the composition of crustal minerals^42^, and is sensitive to the characteristics of fracture, fluid, partial melting and other media^43^. From the vertical profile images in different directions in the Bohai Sea, it can be found that the high value regions of Vp/Vs are mostly concentrated in the top of the mantle, and the Vp of the corresponding regions is also larger. The crust and mantle boundary of the Zhangpeng fault is not a simple interface. It is a transition zone composed of multiple thin layers with a thickness of 6–9 km, which is considered to be the result of material exchange between the crust and mantle^41^. In the Bohai Strait region where earthquakes are concentrated in the Zhangpeng fault (intersection of profiles D and E), the high Vp/Vs value areas in the middle and lower crust are adjacent to the high wave velocity ratio blocks at the top of the mantle. These show that they have similar physical properties, and it is believed that there was once a sign of upwelling of deep thermal material. Some studies have suggested a good correspondence between the strong variation of Vp/Vs structures and the occurrence of earthquakes^17,44^. Through the Vp/Vs model in the Bohai region, it can be found that most relocated earthquakes are distributed in the transitional areas of high and low Vp/Vs values in the crust. These regions are prone to stress concentration due to deformation when the asthenosphere thermal material upwelling, resulting in a large number of earthquakes occurring in the crust above these regions^45^. Therefore, it is considered that the strong seismic activity in the southern Bohai Sea may be related to the deep tectonic extrusion of the upper mantle.

## Conclusion

In this paper, a consistency-constrained double-difference tomography method was used to jointly invert the precise source location parameters and 3D fine structural models of Vp, Vs, and Vp/Vs at depths of over 60 km in Bohai Sea area. This provides important data for understanding the crust and mantle structures and exploring the causes of earthquake formation. Furthermore, the results from this study contribute to promoting research on marine underground structures and enhancing awareness of seismic safety in the sea. The image results show that the relocated earthquakes are mostly distributed at the edges of the high velocity bodies or in the high velocity parts around transition zones between high and low velocity. The velocity structures of P- and S-waves in the Bohai Sea are very similar, with obvious lateral non-uniform characteristics at different depths. Specifically manifested as:

1. The mountainous areas around the Bohai Sea correspond to high-velocity anomalies of the upper crust, while the depressional areas of the surface correspond to low-velocity bodies in the upper crust. The complex crustal structures control the occurrence characteristics of faults and the distribution of earthquakes in the study area. Regarding the obvious crustal stratification structure below the Tanlu Fault, there is a continuous velocity transition in the middle of the crust, with shallow interface depth from south to north, the velocity in the crust gradually increases, and the Vp/Vs shows an obvious high value at the top of the upper mantle. The crustal structure below the Zhangpeng Fault is relatively complex, and there are obvious high-velocity bodies developed in the crust, accompanying low-velocity anomalous areas associated with the top of the mantle in the lower part.
2. The seismic activity in the Bohai Sea is mainly concentrated in the complex velocity structure near the Zhangpeng Fault in the southern Bohai Sea, which is distributed in a high-angle cluster, and the maximum depth reaches the top of the low velocity body in the lower crust. The high Vp/Vs value area near the seismic concentration zone in the southern Bohai Sea is adjacent to the high Vp/Vs block at the top of the mantle. It is considered that there is a close relationship between the upwelling of heat source material in the upper mantle asthenosphere and the seismicity in the crust.
3. The structural difference between the north and south crust is the main feature of the Bohai Strait. There is a low velocity inversion layer in the northern middle crust. The scarcity of seismic activity in the northern part of the Bohai Strait may be attributed to the presence of low-velocity anomalous bodies within the crust. On the contrary, the existence of high-velocity inversion layer in the middle and upper crust of the southern part of the strait, and seismic activity is frequent. The low-velocity layer in the lower crust increases the thickness of the crust-mantle transition zone. The distribution shape of high Vp/Vs value indicates that the low velocity layer at the bottom of the crust is related to the asthenosphere uplift or local heat flow.

## Data Availability

The datasets used in the study can be found online at 10.6084/m9.figshare.24911385.v1.
